# Factors Affecting the Postoperative Bowel Function and Recurrence of Surgery for Intestinal Deep Endometriosis

**DOI:** 10.3389/fsurg.2022.914661

**Published:** 2022-06-14

**Authors:** Ping Xu, Jianzhang Wang, Yanan Zhang, Libo Zhu, Xinmei Zhang

**Affiliations:** Department of General Gynecology, Women’s Hospital School of Medicine Zhejiang University, Hangzhou, China

**Keywords:** intestinal deep endometriosis, segmental resection, disc excision, shaving, postoperative bowel function, complication, recurrence

## Abstract

**Objective:**

This study aims to evaluate the factors associated with complications and long-term results in the surgical treatment of intestinal deep endometriosis and to figure out the optimized treatment measures for bowel endometriosis.

**Methods:**

A retrospective study was performed in a single center in China. Medical charts were reviewed from 61 women undergoing surgical treatment for bowel endometriosis between January 2013 and August 2019 in the Department of General Gynecology, Women’s Hospital School of Medicine Zhejiang University. Multivariate regression analysis was utilized to investigate the impact of the stages of endometriosis and surgical steps (independent risk factors) on complications (and postoperative bowel dysfunction). The clinical characters, surgical procedures, postoperative treatment, complications, and recurrence rate were summarized and analyzed by using Lasso regression.

**Results:**

Surgery type was the most important independent risk factor related to postoperative abnormal defecation in intestinal deep endometriosis patients (*P* < 0.05, OR = 34.133). Infection is the most important independent risk factor related to both postoperative complications (OR = 96.931) and recurrences after conservative surgery (OR = 4.667). Surgery type and age were significantly related to recurrences after conservative surgery.

**Conclusions:**

We recommended conservative operation especially full-thickness disc excision to improve the quality of life of intestinal deep endometriosis patients. In addition, prevention of infection is very important to reduce the postoperative complications rate and the recurrence rate.

## Introduction

Endometriosis is defined as the growth of endometrial cells outside of the uterus. As the second most common gynecological disease, its main symptoms are pain, subfertility, and pelvic mass. Currently, endometriosis is divided into three subtypes, namely, peritoneal endometriosis (superficial endometriosis), ovarian endometriosis, and deep endometriosis (DE). Endometriosis of the bowel can manifest as deeply infiltrative lesions of the muscularis or mucosa or as superficial disease that lines the bowel serosa or subserosal area. It is estimated to affect 3.8%–37% of patients with known endometriosis (1, 2). Such significant differences in the estimated incidence may be due to differences in opinion regarding the definition of intestinal deep endometriosis (IDE) or a reflection of missed diagnosis. IDE is most frequently found on the rectosigmoid colon (90%), followed by the rectum, ileum (12%), appendix (8%), and cecum (6%). The pathogenesis of endometriosis still remains unclear, considering it a multifactorial disorder with possible genetic, hormonal, inflammatory, and immunological causes. Recent studies confirmed that ectopic endometriosis cells needed a pro-inflammatory microenvironment to settle, proliferate, and infiltrate and revealed that patients with endometriosis had an alteration of small bowel permeability, with augmented translocation of lipopolysaccharide (LPS). Then, the transposition of intestinal microbiota and the inflammation of the peritoneal fluid might enhance a chronic low-grade inflammatory state, promoting the sustainable development of endometriosis (3–5). Therefore, more researchers have highlighted the relationships between endometriosis and the host microbiome (6).

In our study, IDE is defined as the lesions of the muscularis or mucosa of the bowel invaded by endometriosis. The incidence of IDE is increasing in recent years, but the treatment of IDE, especially for the optimal surgical approach, is still debated to date, weighing the risks against the benefits. It is disputed whether gynecologists should choose the radical approach like segmental resection, or the conservative approach, such as bowel shaving or full-thickness disc excision (7–10).

The objective of this study was to find out the optimized treatment measures for IDE. We sought to improve the quality of postoperative life and decrease the recurrence of IDE patients. We analyzed the relations between the risk factors and postoperative complications and recurrences of IDE. We showed the recommended surgical approach and key points in the process of clinical treatment of IDE.

## Materials and Methods

This research project was covered by the study approval for data use and clinical studies of the General Ethics Commission, Women’s Hospital School of Medicine, Zhejiang University, China. A retrospective validation analysis was carried out of all patients who underwent IDE operations in the General Gynecologic Department at Women’s Hospital School of Medicine Zhejiang University, from January 2013 to August 2019 (*n* = 61). Four qualified gynecologists performed endometriosis surgery. Interdisciplinary surgery was performed with three experienced colorectal surgeons. Involvement of muscular, submucosal, or mucosal layers were all confirmed by pathology postoperatively (patients presenting with only superficial involvement of bowel serosa were excluded).

Demographics regarding age at the time of surgery, complaints, serum CA125 (cancer antigen 125), localizations of nodules on the digestive tract, nodule size and number, stage of endometriosis according to the revised American Society for Reproductive Medicine (rASRM) score and Enzian classification (11, 12), other associated gynecologic diseases, surgical route, surgical procedures performed, operative time and bleeding, infection after surgery, and the postoperative complications related to each technique were all carefully recorded.

We grouped these 61 patients into 3 different groups, including a shaving group (*n* = 36), disc excision group (*n* = 11), and segmental resection group (*n* = 13, the patient who just had appendectomy was excluded) and summarized clinical characteristics and surgical outcomes of the 3 groups. The shaving group referred to detachment of IDE lesions without penetrating the intestinal cavity (usually intestinal mucosa and deep muscle layer were not involved; the diameter of the lesion was less than 2 cm). The disc excision group referred to the thorough removal of the full thickness of the intestinal wall infiltrated with IDE lesions, followed by a two-layer suture of the defect. The segmental resection group referred to intestinal segment resection with preservation of inferior mesenteric artery and accompanying sympathetic nerves, followed by end-to-end anastomosis, but due to severe adhesion and difficulty in distinguishing pelvic nerves beneath uterosacral ligaments in most endometriosis patients, nerve innervation from inferior hypogastric plexuses could not be totally preserved. Then, we analyzed the risk factors of the major complications (including rectovaginal fistulae and ureteral fistulae), postoperative abnormal defecation (including dyschezia, constipation, frequent defecation, tenesmus, and other discomfort feelings in defecation which differed before operation), infection rate, and recurrence rate. Risk factors included age, CA125, Enzian classification, operative time, infection after surgery, surgery type, and abnormal defecation. After IDE surgery, the drainage tube was usually left in the pelvic cavity and the drainage liquid was always subjected to bacterial culture. Infection after surgery was defined as positive bacterial culture results of the patients’ vaginal discharge, blood, or drainage. According to the result of bacterial culture, body temperatures of patients, and inflammatory biomarkers such as white blood cells, C-reactive protein, and procalcitonin (PCT), sensitive broad-spectrum antibiotics were selected and used for the treatment of the patients with infections, until the body temperature was normal for 3 days.

### Statistical Analysis

Statistical analysis was performed using SPSS 23.0 software (IBM SPSS Statistics). Median values were calculated for continuous variables, and standard deviations or frequencies were calculated for categorical variables. Comparisons of continuous data between the groups were performed using the Kruskal–Wallis *H* test. Fisher’s exact test was applied to compare categorical data between the groups, such as infection rate. STATA 15.0 (StataCorp LLC) and R version 4.0.2 for Windows were applied for statistical analysis. For multivariable analysis, logistic regression and Lasso regression were conducted. The discrimination was quantified with the area under the receiver operating characteristic (ROC) curve. All statistical tests were two-sided, and a *P*-value of <0.05 was considered a significant difference.

## Results

### Comparisons of Clinical Characteristics Stratified by Type of Colorectal Surgery

To compare the treatment effects of IDE patients who underwent different colorectal surgery, we summarized the clinical characteristics and surgical outcomes of the three different groups (Table 1). As to age, operative time, and operative bleeding, there were no significant differences among the three groups. However, we found that the bigger the lesion was, the more radical the surgical procedure was executed (*P* < 0.05). Also, preoperative serum CA125 was much lower in the segmental resection group.

**Table 1:** Comparisons of clinical characteristics stratified by the type of colorectal surgery.

	Shaving (*n* = 36)	Disc excision (*n* = 11)	Segmental resection (*n* = 13)	*P*
Age, year (median, range)	39 (27–52	39 (34–47)	39 (32–47)	0.971
Infertility, *n* (%)	2 (5.6%)	1 (9.1%)	0	
Preoperative serum CA125, U/ml (median, range)	74.5 (13–430)	76.6 (17.4–230.3)	27.2 (7.5–167)	0.032
Diameter of the bowel endometriosis, cm (median, range)	1 (0.5–4)	2 (0.5–3)	4 (1.5–5)	0.000
Operative time, min (median, range)	210 (90–420)	210 (150–480)	240 (150–450)	0.708
Operative bleeding, ml (median, range)	200 (30–1,600)	150 (100–600)	300 (50–1,200)	0.344
Major complication, *n* (%)	2 (5.6%)	2 (18.2%)	0	
Infection after surgery, *n*(%)	2 (5.6%)	4 (36.4%)	3 (23.1%)	0.017
Opening vaginal fornix during surgery, *n* (%)	24 (66.7%)	8 (72.7%)	10 (76.9%)	0.859
Hysterectomy, *n* (%)	11 (30.6%)	1 (9.1%)	7 (53.8%)	0.065
Postoperative abnormal defecation, *n* (%)	2 (5.6%)	1 (9.1%)	8 (61.5%)	0.000
Recurrence, *n* (%)	3 (8.3%)	1 (9.1%)	0	0.625

*One case with only appendix endometriosis was excluded.*

Then, we analyzed the complications among the three groups. Rectovaginal fistulae only occurred after disc excision (*n* = 2), ureteral fistulae only occurred after shaving (*n* = 2), but, notably, ureteral fistulae only occurred in two Enzian stage B3 patients. Ureteral fistulae were more likely to be correlated with huge deep endometriosis mass of the uterosacral ligaments. Postoperative abnormal defecation occurred more frequently in the segmental resection group compared with the shaving group and disc excision group, and the difference was statistically significant (*P* = 0.000, 61.5% vs. 5.6% vs. 9.1%) (Table 1).

The infection rate was significantly lower in the shaving group (*P* < 0.05), compared with the disc excision and segmental resection groups (5.6% vs. 36.4% vs. 23.1%). We speculated that postoperative infection was more likely to occur in the patients with the opening of the vaginal fornix or hysterectomy during surgery. We compared the rate of opening vaginal fornix during surgery between the three groups and found that there was no significant difference (*P* = 0.859). We found that more patients had a hysterectomy in the segmental resection group, compared with the shaving and disc excision groups (53.8% vs. 30.6% vs. 9.1%), but the difference was not significant (*P* = 0.065). Thus, we concluded that the difference in the infection rate among the three groups was correlated with the opening of the bowel lumen rather than the vaginal fornix or hysterectomy.

As to the recurrent rate, although there was no significant difference between the three groups, notably, no recurrent case was found in the segmental resection group, but the recurrent rate was 8.3% and 9.1%, respectively, in the shaving group and disc excision group. Thus, we analyzed the risk factors of the major complications, postoperative abnormal defecation, infection rate, and recurrence rate.

### Conservative Surgery Mitigated Incidence of Postoperative Abnormal Defecation

As reported (13), after conservative surgeries, about 43.8% of the patients still had endometriotic lesions left in the bowel. Even if segmental resection was performed, the histological specimens of about 22% of the patients were found to be positive for the margin. It is very difficult to clear all the lesions in whatever type of surgery patients receive. Therefore, we consider more about the postoperative quality of life of these IDE patients, especially bowel function. Multiple logistic regression analysis was used to determine independent risk factors among associated factors. As shown in Table 2, surgery type was the most important independent risk factor related to postoperative abnormal defecation in IDE patients (*P* < 0.05). Compared with conservative surgery, segmental resection was a risk factor for intestinal dysfunction (OR = 34.133). Other factors, such as age, CA125, size of IDE mass, operative time, and postoperative infection, did not have a significant influence on abnormal defecation (*P* > 0.05). Thus, we concluded that conservative surgery was beneficial to the life quality of the IDE patients when compared to segmental resection. Based on the above data, conservative surgery was recommended for it could mitigate the incidence of postoperative abnormal defecation and improve the life quality of IDE patients.

**Table 2:** Risk factors for abnormal defecation after surgery of intestinal deep endometriosis.

Intercept and variable	*β*	Odds ratio (95% CI)	*P*
Intercept	2.238	–	–
Age	−0.184	0.832 (0.561–1.234)	0.361
CA125	−0.020	0.980 (0.912–1.053)	0.580
Enzian	0.041	1.041 (0.129–8.376)	0.970
Operative time	0.009	1.009 (0.994–1.025)	0.245
Infection after surgery	4.574	96.931 (0.569–16506.920)	0.081
Surgery type	−1.009	0.365 (0.003–51.052)	0.689

*Enzian: classified into C1, C2, and C3 only, indicating the size of IDE lesion; surgery type: classified into disc excision and shaving excision*.

### Infection Was an Important Influencing Factor for the Complications After Conservative Surgery

**Figure 1:**
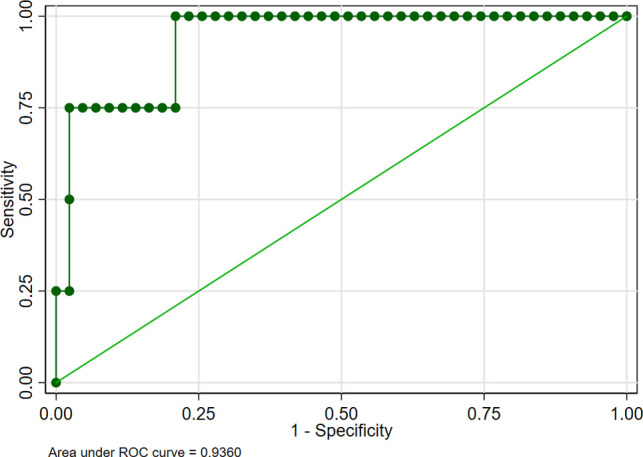
Receiver operating characteristic curve for the predictors of complication after conservative surgery of intestinal deep endometriosis.

**Figure 2:**
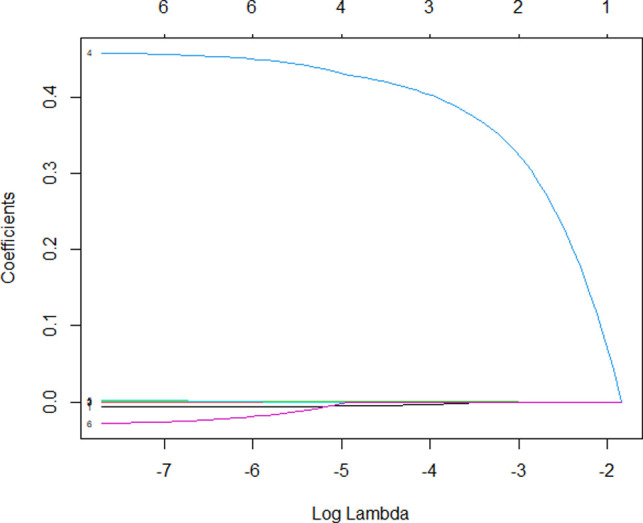
Lasso regression of the predictors for complication after conservative surgery of intestinal deep endometriosis. 4, infection; 6, surgery type.

Although conservative surgery was recommended for IDE, it did not mean that conservative surgery was perfect and complications did not happen after surgery. To make a more targeted analysis of conservative surgery, predictors for complications after conservative surgery of IDE were figured out using multiple logistic regression analysis (Table 3). We showed that infection after surgery was a very strong predictor of complications (OR = 96.931). The area under the curve (AUC) was used to detect postoperative complications via included predictors and the results showed that the AUC was 0.936 (Figure 1), indicating that the included risk factors could effectively predict postoperative complications after surgery of intestinal deep endometriosis. Lasso regression was performed to explore the risk factors related to complications after conservative operations, and the results further showed that infection was the most important influencing factor for postoperative complications (Figure 2) and infection must be controlled after surgery.

**Table 3:** Predictors for complication after conservative surgery of intestinal deep endometriosis.

Intercept and variable	*β*	Odds ratio (95% CI)	*P*
Intercept	6.396	–	–
Age	−0.216	0.806 (0.600–1.083)	0.152
CA125	0.008	1.008 (0.989–1.027)	0.433
Enzian	1.330	3.783 (0.528–27.090)	0.185
Operative time	−0.015	0.985 (0.966–1.005)	0.135
Infection after surgery	1.541	4.667 (0.179–121.571)	0.354
Surgery type	−0.598	0.550 (0.015–19.962)	0.744

*Enzian: classified into C1, C2, and C3 only, indicating the size of IDE lesion; surgery type: classified into disc excision and shaving excision.*

### Infection Was an Important Influencing Factor for the Recurrence After Conservative Surgery

**Figure 3:**
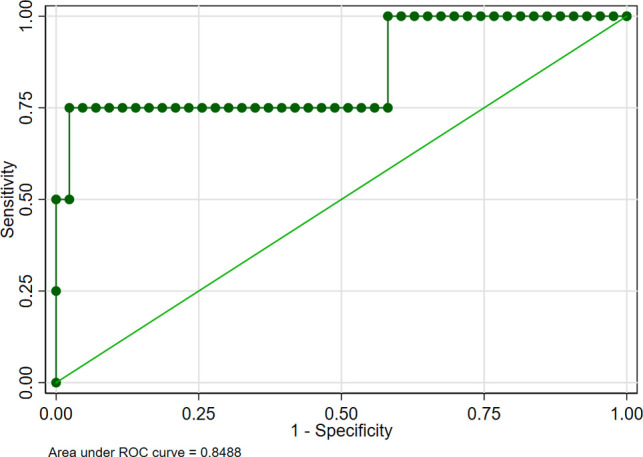
Receiver operating characteristic curve for the predictors of recurrence after conservative surgery of intestinal deep endometriosis.

**Figure 4:**
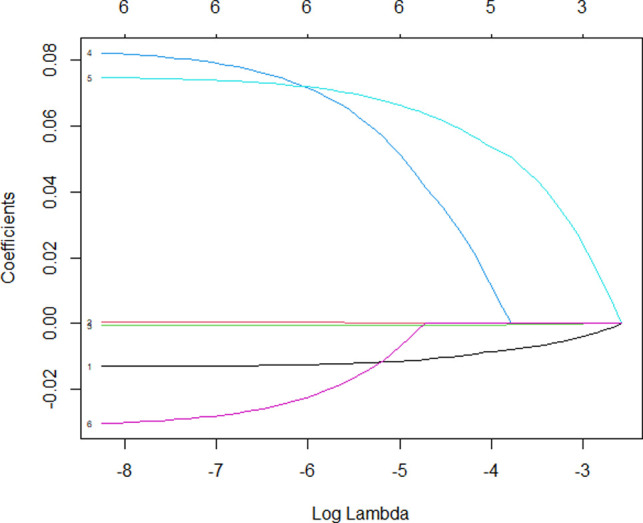
Lasso regression of the included predictors for recurrence after conservative surgery of intestinal deep endometriosis. 1, age; 4, infection; 5, Enzian; 6, surgery type.

In addition to postoperative complications, IDE recurrence was also an important factor related to the life quality of patients and was also a vital criterion to assess whether the surgical procedure was successful or not. Thus, we explored the risk factors of IDE recurrence after conservative surgery. Multivariate logistic regression analysis was performed on the factors related to postoperative recurrence after conservative surgery. We found that infection was the most significant predictor for postoperative recurrence (OR = 4.667, Table 4). In addition to infection, Enzian classification (C1 C2 C3) was another risk factor for postoperative recurrence (OR = 3.783). AUC was utilized to detect the predictive efficacy of factors including postoperative recurrence after conservative surgery. As shown in Figure 3, the AUC was 0.8488, among which infection was the most important influencing factor. Lasso regression of predictors for complication after conservative surgery was performed to further explore the risk factors related to recurrence after conservative operations. As shown in Figure 4, infection and Enzian classification were risk factors for recurrence after conservative operations. We found that age and type of surgery were protective factors for recurrence. Data showed that patients with bowel shaving were more likely to relapse than patients with full-thickness disc excision. Our data indicated that infection was the most important influencing factor for postoperative recurrence and infection must be controlled after surgery, especially in the patients with high Enzian stage (bigger lesion).

**Table 4:** Predictors for recurrence after conservative surgery of intestinal deep endometriosis.

Intercept and variable	*β*	Odds ratio (95% CI)	*P*
Intercept	−5.899	–	–
Age	0.075	1.078 (0.863–1.346)	0.510
CA125	−0.041	0.960 (0.920–1.000)	0.052
Enzian	−0.231	0.794 (0.178–3.536)	0.762
Operative time	0.012	1.012 (0.996–1.028)	0.137
Infection after surgery	−2.445	0.087 (0.003–2.702)	0.163
Surgery type	3.530	34.133 (2.769–420.701)	0.006

*Enzian: classified into C1, C2, and C3 only, indicating the size of IDE lesion; surgery type: classified into segmental resection and conservative surgery*.

## Discussion

IDE is the most severe type of endometriosis, and IDE operation is always one of the most challenging operations for all gynecologists. According to an evidence-based treatment algorithm, Chapron et al. summarized optimal therapeutic treatments for IDE (14). The number, size, depth of the lesion, extent of bowel circumference involvement, and distance to the anal verge would be carefully evaluated before treatment. They suggested that when surgery was chosen, complete resection of endometriosis would be performed, so radical segmental resection was preferred to nodule resections in cases of multiple intestinal nodules, nodules located in the sigmoid, lesions larger than 3 cm in size, and deep lesions involving the submucosa and/or mucosa. However, they compared digestive function only before and after surgery, rather than between three different surgical procedures. We proposed that physicians should consider improving postoperative life quality and reducing recurrence when selecting treatment options. Remarkably, as opposed to segmental resection, conservative procedures had fewer intestinal dysfunctional symptoms, such as dyschezia, constipation, or frequent defecation. The postoperative dysfunction of the bowel in patients receiving segmental resection may be related to the extensive dissection of the retrorectal space with disruption of the surrounding neurovascular structures, such as superior and inferior hypogastric plexus, as well as the sympathetic and parasympathetic nerve bundles, causing short- and long-term morbidity (9, 15–17). Thus, gynecologists and surgeons have focused on the nerve-sparing techniques in IDE operations, and the pelvic nerve-sparing technique and preservation of the inferior mesenteric artery (with ligation of the sigmoid arteries one by one near the bowel wall) have been proposed to reduce intestinal denervation and improve postoperative digestive, urinary, and sexual functions (18, 19). With the precise protection of the inferior hypogastric plexuses and companion nerves of the inferior mesenteric artery, no matter what surgical procedure is executed, functional preservation can be assured. Anorectal manometry showed no reduction in rectoanal inhibitory reflex and hypertone of the internal anal sphincter before and after nerve-sparing surgery of the posterior DE nodule (20).

Also, in our study, although the recurrence was less in the segmental resection group, there was no significant difference in recurrent rate among the three groups. Mabrouk et al. also found that the recurrent rate showed no significant difference, and there were no significant differences in the rate of chronic constipation and urinary retention as well. However, the segmental resection group had more short-term complications. Therefore, they preferred conservative surgery rather than radical surgery in IDE patients with median lesions (21). Remorgida et al. discovered that even with radical segmental resection, occult microscopic endometriosis has been shown to be present in 15% of specimen resection margins (22). In this study, we had the same conclusion as these researchers, presenting that radical surgery, therefore, may not improve overall long-term outcomes as compared with conservative surgery, yet it is linked to a higher risk of bowel dysfunction (23). Therefore, we would recommend conservative surgeries in IDE with longtime postoperative drug usage.

As to conservative operative types, full-thickness disc excision is a protective factor for IDE recurrence compared with bowel shaving. Patients with full-thickness disc excision may have high-quality life than patients with bowel shaving. However, our data showed that patients with full-thickness disc excision have a higher infection rate than patients with bowel shaving. In addition, we found that conservative operations were more likely to have major complications than the segmental resection group. To avoid major complications such as intestinal fistula and reduce infection rate, we have been working to improve the surgical approach of full-thickness disc excision.

The vital points of the procedure are as follows: (1) thorough excision of the lesion to ensure that the suture site was normal tissue to get better healing and lower the possibility of intestinal fistula and (2) sufficient dissociation of the bowel to avoid tension on the suture. We suggest that mobilization of the rectum is carried out at least 20 mm below the rectal nodule. (3) Sufficient blood supply of the bowel wall needs to be ensured, (4) a two-layer suture technique rather than a one-layer suture is strongly recommended, (5) a rectal air test is indispensable to check the integrity and avoid leakage, (6) fasting to unobstructed exhaust after the operation, and (7) application of sufficient broad-spectrum antibiotics for intestinal Gram-negative bacteria. That is because the intestinal lumen is opened, leading to a higher risk of contamination, postoperative infection, and then severe complications such as intestinal fistula.

In the last decade, many efforts have been made to reduce possible complications in IDE operations. Endovenous indocyanine green (ICG) was proposed to assess the perfusion of the bowel, with an ischemic area indicating fibrotic endometriotic lesion, and select the transecting line. Furthermore, with ICG fluorescence imaging, the blood supply to the anastomosis can be objectively evaluated to prevent intestinal fistulae (24). However, it is still unclear for the precise reason for complications. Therefore, it deserves further investigation and we found that infection was an important influencing factor for the complications after conservative surgery. In our study, postoperative infection increased the incidence of postoperative difficulty in defecation and increased the risk of postoperative recurrence. Therefore, we believe that prevention and treatment of postoperative infection should be taken as an important consideration in the management of IDE patients. Thus, we concluded that the crucial therapeutic strategy is infection prevention, including mechanical bowel preparation (25, 26) and intraluminal antibiotic decontamination of the bowel before surgery, rational use of antibiotics in the perioperative period, strict sterilization, thorough povidone-iodine solution flush during surgery, and postoperative broad-spectrum antibiotic usage.

However, our research had some limitations. It would have been better to evaluate the comparison of all parameters with the same size of the intestinal nodule. The sample number was relatively small. This limitation affected the analysis of risk factors for complications and recurrence. We will continue with this study and enlarge the sample number. We hope our study will serve as a better reference for the selection of IDE treatment regimens in the future.

## Conclusion

Our study provides a reference for the selection of IDE surgical protocols and explores the key factors in the clinical treatment of IDE. Conservative operation was recommended for IDE to improve the quality of life of IDE patients. In addition, prevention of infection is very important to reduce the postoperative complications rate and the recurrence rate.

## Data Availability

The raw data supporting the conclusions of this article will be made available by the authors, without undue reservation.
